# Ephedrine vs. phenylephrine effect on sublingual microcirculation in elderly patients undergoing laparoscopic rectal cancer surgery

**DOI:** 10.3389/fmed.2022.969654

**Published:** 2022-10-05

**Authors:** Yanbing Zhang, Limin Jin, Huayue Liu, Xiaowen Meng, Fuhai Ji

**Affiliations:** Department of Anesthesiology and Pain Management, The First Affiliated Hospital of Soochow University, Suzhou, China

**Keywords:** ephedrine, phenylephrine, sublingual microcirculation, laparoscopic rectal cancer, surgery

## Abstract

**Background:**

The effects of anesthesia administration on sublingual microcirculation are unknown. It is unclear how sublingual microcirculation responds to ephedrine or phenylephrine administration. We hypothesized that microvascular perfusion is impaired under anesthesia.

**Materials and methods:**

We randomly divided 100 elderly patients undergoing laparoscopic rectal cancer surgery into phenylephrine and ephedrine groups in a 1:1 ratio. Ephedrine or phenylephrine was administered when MAP was < 80% for > 1 min. The heart rate (HR) and mean arterial pressure (MAP) were recorded every 5 min. Lactic acid was tested both pre- and postoperatively. The sublingual microcirculation characteristics of the microvascular flow index, the percentage of perfused vessels, the density of perfused vessels, and the heterogeneity index were monitored using a sidestream dark field imaging device.

**Results:**

Their MAP showed an evident decrease of > 20%. At this point, the HR, microvascular flow index, perfused vessel density, and proportion of perfused vessels decreased similarly in ephedrine and phenylephrine groups. Conversely, the heterogeneity index increased in both groups. After phenylephrine and ephedrine administration, ephedrine treatment significantly increased the proportion of perfused vessels, microvascular flow index, and HR compared with phenylephrine treatment.

**Conclusion:**

General anesthesia was associated with reduced MAP, HR, and sublingual microcirculation in elderly patients undergoing laparoscopic rectal cancer surgery. The results of ephedrine treatment were better than those of phenylephrine treatment in terms of HR, increased the proportion of perfused vessels, and microvascular flow index of sublingual microcirculation.

**Clinical trial registration:**

[www.ClinicalTrials.gov], identifier [ChiCTR-2000035959].

## Introduction

Intraoperative hypotension is among the most common complications presenting during non-cardiac surgery (1). Most patients experience at least one episode during which the mean arterial pressure (MAP) falls below 65 mmHg, and this typically happens shortly after inducing anesthesia (2). Intraoperative hypotension can lead to highly unfavorable postoperative outcomes, such as acute kidney injury, death, and myocardial infarction (3, 4). Recently, in a randomized controlled trial, Hamilton et al. reported that the risk of postoperative organ dysfunction approximately reduces by 25% if intraoperative hypotension is prevented (5). Particularly, elder patients undergoing surgery for rectal cancer generally develop poor vascular compliance, high blood pressure, and low blood volume intraoperatively. In high-risk surgical patients, postoperative outcomes are reportedly improved by perioperative hemodynamic optimization (6), which reportedly reduced postoperative complications, length of stay, and mortality (7, 8). In the present study, during rectal surgery, our anesthesiologist had to maintain adequate perfusion pressure so that sufficient blood flow to the heart and brain is ensured for meeting metabolic demands. The anesthesiologist also had to eliminate any chances of adverse emergency response to the surgery. General anesthesia induction typically reduces the cerebral perfusion pressure and MAP; this can be attributed to systemic vascular resistance and decreased cardiac output, particularly in the elderly (9). To treat anesthesia-related hypotension and counteract cardiovascular depression, ephedrine, which is an indirectly acting β- and α-adrenergic agonist, and phenylephrine, which is a pure α-adrenergic agonist, are typically given during rectal operations (9). With the use of vasopressors, vascular resistance is increased and cerebral perfusion pressure improved; however, while they do improve the blood supply, they may inadvertently impair oxygen extraction from the blood by causing capillary flow pattern disturbances, and this may happen even if the target cerebral perfusion pressure and perfusion of other organs (e.g., the kidney) have been achieved (10–12).

Little is known on whether ephedrine or phenylephrine affect sublingual microcirculation in the elderly undergoing laparoscopic surgery for rectal cancer. Microcirculation monitoring can be done for few sites with easy access. A handheld vital microscope, which is a sidestream dark field imaging device, can be used to obtain sublingual microcirculation sequences. It is a non-invasive technique and an effective tool to assess microvascular variables like heterogeneity index, percentage of perfused vessels, microvascular flow index, and perfused vessel density (13).

Microvascular perfusion alterations can lead to mortality and organ dysfunction in hemorrhagic and septic shocks (14–17). The associations between microcirculation and macrovascular hemodynamic optimization in the state of a shock have been explored by several research groups (13, 18). Few studies have analyzed the behavior of microcirculation in epidural patients (19) and cardiopulmonary bypass operation (20). However, in the elderly, microcirculation changes during rectal cancer operation have been seldom analyzed.

The present study was aimed at testing the hypothesis that microvascular perfusion is impaired during laparoscopic rectal cancer surgery in elderly patients and that it recovers after the administration of phenylephrine and ephedrine.

## Materials and methods

### Ethics

After obtaining approval from the Ethical Review Board of our hospital (The First Affiliated Hospital of Soochow University No. 2019130), 100 patients were recruited to this study; all patients provided written informed consent. This clinical trial was registered by one of the study investigators (Fuhai ji, 2020-21-08) in the Chinese Clinical Trial Registry (registration no., ChiCTR2000035959).

### Trial design

This was a prospective parallel-group, double-blinded, single-center, enrolled and randomized trial.

### Patients and setting

All patients aged 65–85 years with an American Society of Anesthesiologists grade of I–II who were scheduled for laparoscopic surgery for rectal cancer were screened. The exclusion criteria were as follows: (1) Loose teeth, oral damage or bleeding, difficulty in opening the mouth, and challenges with performing sublingual microcirculation monitoring; (2) Hb < 90 g/L or Hct < 25%, abnormal blood coagulation; (3) sinus arrhythmia or a New York Heart Association (NYHA) grade ≥ II; (4) patients within 3 months undergoing second surgery; and (5) refusal to provide written informed consent. If patients had a heart or brain disease, they were considered to have NYHA grade ≥ II. renal function failure, an American Society of Anesthesiologists Physical Status III–V, and a history of allergy to one of the study medications were excluded. In line with the non-interventional study design, sublingual microcirculation was examined with a completely non-invasive sidestream dark field imaging device (MicroSee, V100, Guangzhou Medsoft System).

#### Study protocol

Using a random number sheet generated by a computer software (Microsoft Excel, Redmond, Washington, USA), patients were allocated into one of the two groups. The random number sheet was placed in an opaque envelope by an anesthetist (MXW) before the clinical trial was initiated. The patients in two groups received ephedrine and phenylephrine infusion. The study drug, which was diluted to 50 mL in identical 50-mL infusion syringes under sterile conditions, was prepared by an anesthetist (MXW) who was not involved in managing the patients’ hemodynamics.

### Macrocirculation monitoring

On the day the surgery was scheduled, an intra-arterial catheter was placed in the radial artery in patients to obtain arterial blood gas samples and continuous blood pressure measurements. Cisatracurium, propofol, and sufentanil were used as anesthesia induction agents, followed by sevoflurane administration, which was used for maintenance. Intraoperative analgesia was ensured with intravenous remifentanil. Sodium Potassium Magnesium Calcium and Glucose Injection is infused at a rate of 4 mL/kg/h. Local care protocols were followed for treating all patients. The following parameters were collected and recorded at 5-min intervals on the monitoring report: the MAP, heart rate (HR), respiratory rate, pulse oxygen saturation, bispectral index, temperature measured from the Philips IntelliVue Monitoring (Philips Medical Systems, USA), carbon dioxide, inspired and expired oxygen, tidal volume (VT), and volatile anesthetic fractions.

### Sublingual microcirculation monitoring

The sidestream dark field imaging device was used to obtain videos of sublingual microcirculation. This device works polarized spectral imaging technology-derived technique (21). These videos allowed us to directly visualize sublingual blood flow inside the microvascular networks. The emitted light corresponded to the wavelength absorbed by hemoglobin, and each erythrocyte was displayed in black on a light background. Image acquisition and image analysis were performed in compliance with international guidelines. Based on published recommendations, a lot of care was taken to avoid pressure artifacts. Massey et al. had described the “microcirculation image quality score,” which was used for systematic evaluation of the sequence quality (12). Next, MicroSee Information Management Software was used to complete the evaluation (13). Sequences with poor quality were eliminated at bedside itself. Furthermore, to ensure the availability of two good quality sequences for each measurement point, another round of sequence acquisition was performed. Herein, a blinded analysis was facilitated by concealing patient identity as well as video identification information and replacing these identifiers with a randomly assigned alphanumeric code.

### Clinical management

Patient randomization ensured 1:1 distribution and was done such that the patients received the infusion of either ephedrine (6 mg/mL) or phenylephrine (0.05 mg/mL). When the MAP fell below 80% of the baseline value or 65 mmHg for more than 3 min, ephedrine or phenylephrine was administered. Herein, it was administered using a target-controlled infusion for maintaining stable perioperative blood pressure after a bolus injection. Phenylephrine infusion rate was 1 μg/kg/min, and ephedrine infusion rate was 80 μg/kg/min. If a patient’s HR fell below 50 beats/min, 0.3–0.5 mg atropine was used to normalize it. Regarding the ventilator settings, V_*T*_ was 8 mL/kg^–1^ of the ideal body weight; moreover, the respiratory rate was set such that the end-tidal carbon dioxide was kept between 36 and 45 mmHg.

#### Sample size estimation

Sample size was calculated with the Cochran–Armitage Test using Power Analysis and Sample Size (PASS version 11.0.7; NCSS, LLC, Kaysville, Utah, USA). Calculations were based on the early preliminary data that showed that the proportion of patients experiencing hypotension for more than 1 min during anesthesia induction was 82%. Using a *Z-*test with continuity correction, we determined that a sample size of 88 patients in total (44 patients per group) would have 90% power to detect a linear trend in the proportion of patients with hypotension among groups with a significance level of 0.05. Considering the possibility of dropouts, the sample size was increased to 100 patients.

### Statistical analysis

Changes in macrovascular and microvascular parameters were evaluated both before and after anesthesia administration. These parameters varied over time, and these changes in microvascular parameters (heterogeneity index, microvascular flow index, percentage of perfused vessels, and perfused vessel density) and macrovascular parameters (HR and MAP) were analyzed by drawing comparisons of the values associated with the time of ephedrine or phenylephrine administration; furthermore, the time points were recorded using the Wilcoxon signed-rank test for microvascular parameters showing non-Gaussian distribution or using *t*-test for matched pairs for macrovascular parameters showing Gaussian distribution (macrovascular parameters).

## Results

### Demographic data

This study was conducted from 19 October 2019 to September 2020. Herein, 100 patients were enrolled. Five patients were administered neither phenylephrine nor ephedrine because changes in their MAP after inducing anesthesia were non-compliant with the predefined criteria. Furthermore, in four patients, strong signal interference made image analysis impossible, thus leading to their exclusion. Moreover, oral and written informed consent was provided by each patient before participation in the study. The mean age of the included patients was 72.2 years, and the mean New York Heart Association score was 1. Table 1 shows the patients’ demographic data.

**TABLE 1:** Patient characteristics during surgery in ephedrine and phenylephrine groups.

	Group		Statistic	*P*
	E (*n* = 45)	P (*n* = 46)		
Age (years)	71.00 (70.34–73.48)	70.00 (70.56–74.31)	1033.5^a^	0.990
Sex (M/F) High (cm)	31/14 163.82 ± 9.17	32/14 162.04 ± 8.78	0.005 0.946	1.000 0.347
Weight (kg)	64.93 ± 9.33	62.30 ± 12.05	1.162	0.248
Hypertension	35/10	36/10	0.003^b^	0.956
AST (IUl-1)	18.00 (17.46–21.83)	16.85 (16.4–21.72)	935^a^	0.427
Plasma lactate (mmol l^–1^)				
Start of surgery	0.67 ± 0.10	0.648 ± 0.11	–1.739	0.09
End of surgery	0.71 ± 0.13	0.68 ± 0.11	–1.252	0.22
Operative duration (min)	116.89 ± 9.31	116.04 ± 11.39	0.313	0.755
Crystalloid (mL)	548.8 ± 48.8	540.00 ± 56.52	0.802	0.425
Volume (mL)	494 ± 46.88	505.22 ± 6759	–0.927	0.356

^a^Mann–Whitney test, ^b^Chi-square test. ASA, American Society of Anesthesiologists; MAP, mean artery pressure; AST, glutamic-oxaloacetic transaminase.

Data for categorical variables are presented as frequency (%) and for continuous variables as means ± SD. The *P*-value < 0.05 denotes the statistical difference between the phenylephrine group and the ephedrine group.

### Study flow

**FIGURE 1:**
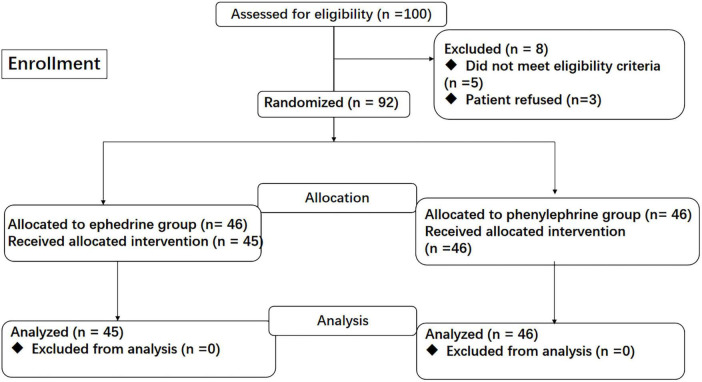
CONSORT flow chart.

A total of 100 patients were screened for eligibility. Of 8 patients who were excluded after screening, 5 patients did not meet the eligibility criteria (5 with Surgical approach changed), and 3 patients declined to participate. 1 patient withdrew the consent before anesthesia. Finally, 91 patients completed this study (ephedrine, *n* = 45; phenylephrine, *n* = 46; Figure 1).

### Baseline value measure macrovascular and microcirculation parameters

We evaluated the microcirculatory baseline of the elder individuals with the same characteristics 5 min before anesthesia. In ephedrine group, the base value of MAP baseline was 107.56 mmHg ± 11.62 mmHg vs. 109.17 mmHg ± 14.84 mmHg in phenylephrine group. HR baseline was 73.44 bpm ± 8.64 bpm vs. 77.54 bpm ± 14.56 bpm in phenylephrine group. The base value of microcirculation parameters in ephedrine group was MFI 2.85 ± 0.26, PPV 98.71 ± 3.03, PVD 16 ± 3.78 and HI was 0.17 ± 0.31 vs. MFI 2.87 ± 0.21, PPV 99.16 ± 2.55, PVD 16.33 ± 4.54 and HI was 0.1 ± 0.19 in phenylephrine group (*P* > 0.05).

### Changes in macrovascular variables during the induction period

**FIGURE 2:**
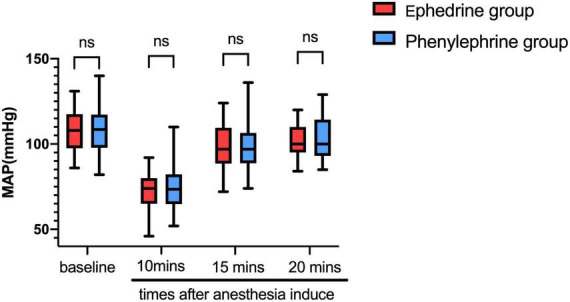
Ephedrine and phenylephrine administration were consequently performed as per the protocol. MAP showed evident changes in both groups (in this Figure). VC, vasoconstrictor.

During anesthesia induction, 91 patients experienced an episode of low blood pressure. Furthermore, the MAP was significantly reduced by general anesthesia. Ephedrine and phenylephrine administration were consequently performed as per the protocol. MAP showed evident changes in both groups (Figure 2).

**FIGURE 3:**
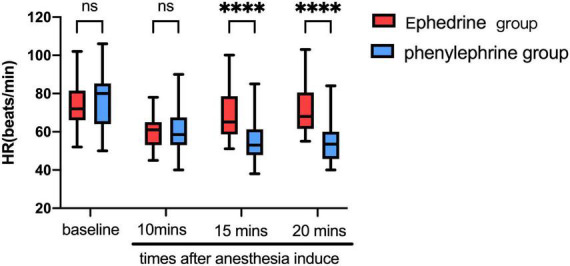
The ephedrine group showed increased HR 5 min after ephedrine injection from 57.20 bpm ± 7.99 bpm to 60.37 bpm ± 10.43 bpm, whereas the phenylephrine group showed decreased HR from 60.37 bpm ± 10.43 bpm to 55.61 bpm ± 11.53 bpm. (VC, vasoconstrictor; represent ephedrine or phenylephrine) (^∗∗∗∗^*P* < 0.0001).

**FIGURE 4:**
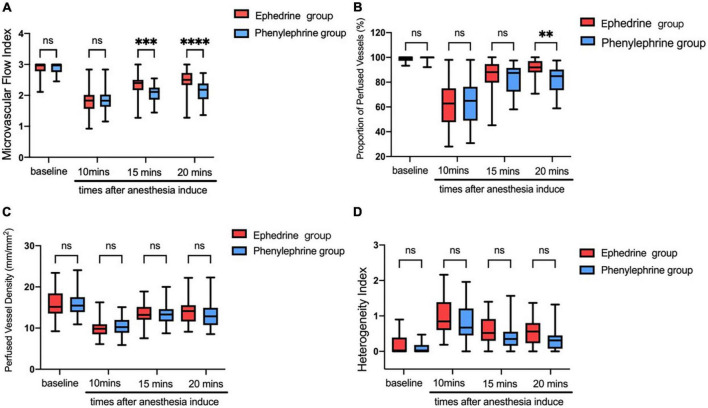
**(A)** The microvascular e flow index showed evident decrease after anesthesia induction in both groups. The ephedrine group showed better microvascular flow index than the phenylephrine group (^∗∗^*P* < 0.01, ^∗∗∗^*P* < 0.001, ^∗∗∗∗^*P* < 0.0001). **(B)** The proportion of perfused vessels showed evident decrease after anesthesia induction finished in both groups. Both groups showed evident increases after VC injection (^∗∗^*P* < 0.01). **(C)** The perfused vessel density showed evident decreases after anesthesia induction finished in both groups. Both groups showed evident increases after VC injection. **(D)** The proportion of perfused vessels showed evident decreases after anesthesia induction in both groups. Both groups showed obvious increases after VC injection.

In the ephedrine group, propofol, sufentanil, and cisatracurium were used as anesthesia induction agents. At an average of 10 min after anesthetic drug administration, the MAP declined from 107.56 mmHg ± 11.62 mmHg at baseline to 71.16 mmHg ± 10.51 mmHg (*P* < 0.01), whereas the HR declined from 73.44 bpm ± 8.64 bpm at baseline to 57.20 bpm ± 7.99 bpm (*P* < 0.01). Then, 5 min after ephedrine administration, the MAP increased to 98.02 mmHg ± 12.96 mmHg. Moreover, 10 min after ephedrine administration, the MAP increased to 102.04 mmHg ± 10 mmHg, and the HR increased from 57.20 bpm ± 7.99 bpm to 60.37 bpm ± 10.43 bpm (Figures 3, 4). In the phenylephrine group, at an average of 10 min after propofol, sufentanil, and cisatracurium administration, the MAP declined from 109.17 mmHg ± 14.84 mmHg at baseline to 73.98 mmHg ± 12.13 mmHg, and the HR declined from 77.54 bpm ± 14.56 bpm at baseline to 60.37 bpm ± 10.43 bpm. Then, 5 min after phenylephrine administration, the MAP increased to 98.02 mmHg ± 14.74 mmHg, and the HR decreased from 60.37 bpm ± 10.43 bpm to 55.61 bpm ± 11.53 bpm. Then, 10 min later, the MAP increased from 73.98 mmHg ± 12.13 mmHg to 102.04 mmHg ± 10 mmHg, and the HR decreased to 54.67 bpm ± 11.01 bpm (Figures 2, 3) (VC: vasoconstrictor).

### Changes in microvascular variables during the anesthesia period

In the ephedrine group, propofol, sufentanil, and cisatracurium were used as anesthesia induction agents. Herein, 10 min after drug administration, a decrease in the microvascular flow index was noted from 2.85 ± 0.23 at baseline to 1.86 ± 0.39 at the time of ephedrine injection (Figure 4A); at the same time, the percentage of perfused vessels reduced from 98.71% ± 2.19% to 61.37% ± 17.87% (Figure 4B), and the density of perfused vessels dropped from 15.97 ± 3.19 mm/mm^2^ to 10.04 ± 2.17 mm/mm^2^ (Figure 4C). An increase in the heterogeneity index was noted from 0.17% ± 0.25% to 0.99% ± 0.46% (Figure 4D). Five minutes after ephedrine injection, the microvascular flow index increased to 2.43 ± 0.42, density of perfused vessels increased to 13.5 ± 3.01 mm/mm^2^, the percentage of perfused vessels increased to 85.17% ± 13.02%, and heterogeneity index declined to 0.61% ± 0.47%. Ten minutes later, the percentage of perfused vessels increased to 90.23% ± 14.17%.

In the phenylephrine group, the microvascular flow index decreased from 2.89 ± 0.16 at baseline to 1.85 ± 0.36 at the time of phenylephrine injection (Figure 4A), and at the same time, the percentage of perfused vessels reduced from 99.16% ± 1.74% to 64.02% ± 16.05% (Figure 4B), and the perfused vessel density dropped from 16.33 ± 4.24 mm/mm^2^ to 10.37 ± 2.12 mm/mm^2^ (Figure 4C). An increase in the heterogeneity index was noted from 0.10 ± 0.14 to 0.83 ± 0.52; 5 min after phenylephrine injection (Figure 4D), the microvascular flow index increased to 2.07 ± 0.37, density of perfused vessels increased to 13.33 ± 3.34, the percentage of perfused vessels increased to 82.34% ± 12.07%, and heterogeneity index declined to 0.39% ± 0.41%.

#### Correlation analysis

**FIGURE 5:**
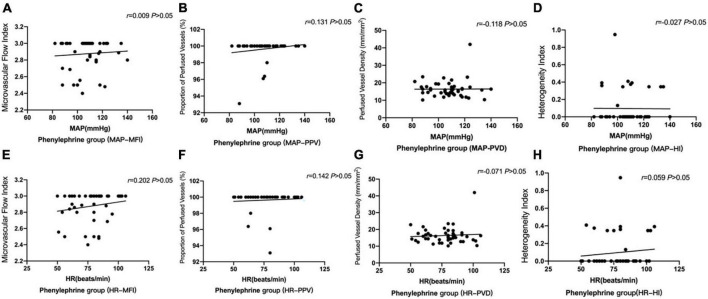
Correlation of MAP with microcirculation parameters in the ephedrine group **(A–D)**. Correlation of HR with microcirculation parameters in the ephedrine group **(E–H)**. **(A)** The correlation of MAP with MFI in the ephedrine group (*P* > 0.05). **(B)** The correlation of MAP with PPV in the ephedrine group (*P* > 0.05). **(C)** The correlation of MAP with PVD was negative in the ephedrine group (*P* < 0.05). **(D)** The correlation of MAP with HI in the ephedrine group (*P* > 0.05). **(E)** The correlation of HR with MFI in the ephedrine group (*P* > 0.05). **(F)** The correlation of HR with MFI in the ephedrine group (*P* > 0.05). **(G)** The correlation of HR with MFI in the ephedrine group (*P* > 0.05). **(H)** The correlation of HR with MFI in the ephedrine group (*P* > 0.05).

We analyzed the correlation of MAP with the microcirculation parameters MFI, PVD, PPV, and HI in the ephedrine group; MAP was found to have no correlation with MFI, PPV, and HI (Figures 5A,B,D). However, MAP was negatively correlated with PVD in the ephedrine group (Figure 5C) (*P* < 0.05).

Meanwhile, we analyzed the correlation of HR with the microcirculation parameters MFI, PVD, PPV, and HI. Consequently, HR was found to have no correlation with MFI, PPV, and HI (Figures 5E–H) (*P* > 0.05).

**FIGURE 6:**
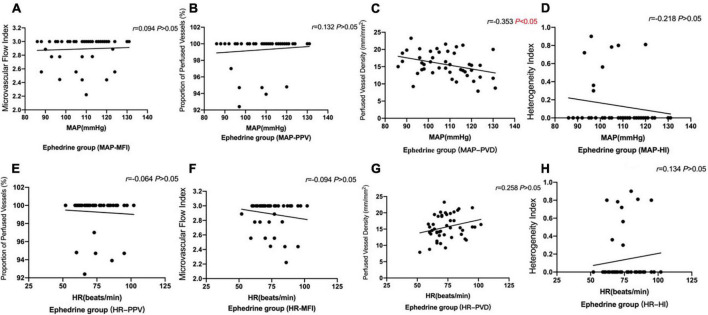
The correlation of MAP and HR with the microcirculation parameters MFI, PVD, PPV, and HI in the phenylephrine group. MAP was found to have no correlation with microcirculation parameters figures. **(A)** The correlation of MAP with MFI in the phenylephrine group (*P* > 0.05). **(B)** The correlation of MAP with PPV in the phenylephrine group (*P* > 0.05). **(C)** There is no correlation MAP with PVD in the phenylephrine group (*P* > 0.05). **(D)** There is no correlation MAP with HI in the phenylephrine group (*P* > 0.05). **(E)** There is no correlation HR with MFI in the phenylephrine group (*P* > 0.05). **(F)** There is no correlation HR with PPV in the phenylephrine group (*P* > 0.05). **(G)** There is no correlation HR with PVD in the phenylephrine group (*P* > 0.05). **(H)** There is no correlation HR with HI in the phenylephrine group (*P* > 0.05).

We analysis the correlation of MAP with the microcirculation parameters MFI, PVD, PPV and HI in the phenylephrine group. MAP was found to have no correlation with MFI, PPV, PVD, and HI (Figures 6A–D; *P* > 0.05).

Meanwhile, we analyzed the correlation of HR with the microcirculation parameters MFI, PVD, PPV, and HI, and consequently, HR was found to have no correlation with MFI, PPV, and HI (Figures 6E–H; *P* > 0.05).

### Sublingual microcirculation images

**FIGURE 7:**
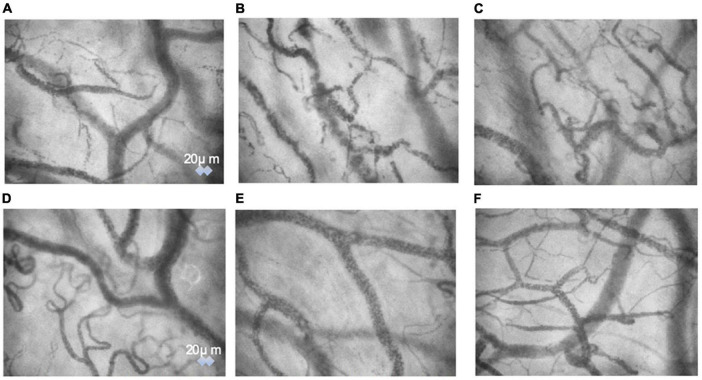
The sidestream dark field images obtained through the videos of sublingual microcirculation. We have provided three images showing patients’ base state **(A)**, 10 min after anesthesia induction **(B)**, and 5 min after ephedrine administration **(C)**. The images for the phenylephrine group at the same time points have been provided as well **(D–F)**.

The sidestream dark field images obtained through the videos of sublingual microcirculation. We have provided three images showing patients’ base state (Figure 7A), 10 min after anesthesia induction (Figure 7B), and 5 min after ephedrine administration (Figure 7C). The images for the phenylephrine group at the same time points have been provided as well (Figures 7D–F).

## Discussion

Our study comprehensively assesses sublingual microcirculation in elderly patients undergoing laparoscopic rectal cancer surgery and quantitatively depicts its heterogeneity, perfusion, and density components. The primary finding of our study was that elderly patients exhibited evident sublingual microcirculatory alterations accompanied with reduced MAP and HR. The disorder presented with decreases in the presence of capillaries with hyperdynamic flow, proportion of perfused vessels, and vascular density. We used ephedrine and phenylephrine to address hypertension. We found that phenylephrine caused more significant reduction in HR. The ephedrine group showed a higher proportion of perfused vessels for sublingual microcirculation than the phenylephrine group. The latter was better than the former in reducing the heterogeneity index parameters.

Hypotension is frequently encountered intraoperatively, particularly during general anesthesia induction. However, studies use widely diverse criteria for intraoperative hypotension (22, 23). Both absolute (e.g., MAP < 65 mmHg) and relative (e.g., MAP reduction, ≥ 20% relative to baseline) thresholds have been used to define intraoperative hypotension. In our research, we defined hypotension with the thresholds of MAP reduction ≥ 20% relative to baseline. In older patients, since systemic hemodynamics have range change and the characteristics are different from young people when under general anesthesia, we herein hypothesized that the sublingual mucosa in such patients can reveal microcirculatory abnormalities. Verdant et al. found that among ambulatory adults aged ≥ 75 years, treating to an SBP target of less than 120 mm Hg compared with an SBP target of less than 140 mm Hg resulted in significantly lower rates of fatal and non-fatal major cardiovascular events and death from any cause (24).

This study shows that sublingual microcirculation disorder in patients undergoing laparoscopic rectal cancer surgery was associated with anesthesia induced hypertension. However, our findings show that sublingual microcirculation parameters were not positively correlated with MAP and HR, and interestingly, MAP was negatively correlated with PVD. Our findings suggested reduced sublingual microcirculation was accompanied with blood pressure decline in these patients. However, after MAP decline, we noted impaired microvascular perfusion during anesthesia. Adrenergic agonists like ephedrine or phenylephrine were used to increase MAP and consequently found microvascular perfusion to recover with both treatments.

Indeed, some sublingual microcirculatory variables, such as the percentage of perfused vessels, the perfused vessel density, and the microvascular flow index, significantly decreased alongside MAP decline. However, both ephedrine and phenylephrine restored the MAP and microvascular variables. Notwithstanding, it is notable that they differently affected the HR and the degree of sublingual microvascular variables. After administering ephedrine and phenylephrine, evident increases in the microvascular flow index, MAP, the percentage of perfused vessels, and the perfused vessel density were noted in both groups; conversely, HR showed an evident decline in the phenylephrine group. In a prior study of 255 patients, which defined hypotension as a MAP of < 65 mmHg, 87% of the patients intraoperatively experienced ≥ 1 min hypotensive episode (22). Our experimental results are similar.

For the elderly patients, propofol- and sufentanil-induced hypotension is caused by a decrease in sympathetic activity caused by these agents. This decrease includes decreases in cardiac output and in systemic vascular resistance caused by the combined effect of depression of myocardial contractility, venous and impaired baroreflex, and mechanism arterial vasodilation. We observed increases in the MAP after phenylephrine and ephedrine treatments as well as simultaneous increases in the PPV and microvascular flow index. The real mechanism still remains unknown.

Although only sublingual microcirculation was identified in the current study, many studies have demonstrated a correlation of sublingual microcirculatory changes with gut (24–26), and renal microcirculatory changes (27, 28).

In the present study, we assessed the characteristics of sublingual microcirculation in patients undergoing laparoscopic rectal cancer surgery. Ephedrine and phenylephrine are typically used to maintain or restore oxygen delivery to the tissues. Predicting microcirculation patterns solely based on the changes in macrocirculatory variables is challenging. Indeed, considering the decreased cardiac output, which was followed by decreases in the MAP, the physiologic microvascular response seems primarily aimed at preserving tissue perfusion. It should be noted, however, that altered microcirculation during shock is still possible despite the optimization of macrocirculation (29). This finding is because microcirculation is altered in specific ways when in shock and exhibits glycocalyx or erythrocyte alterations, viscosity changes, and endothelial dysfunction (30). There could be variations in the degree of microcirculatory alterations; it may vary over time as well as with the difference in the intensities of inflammation and shock. In this regard, in early hypotension, improvements in microcirculation were observed after ephedrine and phenylephrine treatments, which is notable since persistent hypotension can cause inevitable injury to vital organs. In the present study, the assessment of sublingual microcirculatory variables was performed in patients with prior microvascular impairment and hemodynamic instability.

Our study suggests that anesthesia induction with sufentanil and propofol impacts sublingual microcirculation (and potentially microcirculation in other sites as well) in patients undergoing colorectal cancer surgery. It should be noted that predicting microcirculation patterns solely based on changes in macrocirculatory variables is challenging. For the MAP to reach the baseline levels, the microcirculation characteristics of microvascular flow index not recovered to baseline level.

One interesting finding of our study was that the HR level was evidently different between phenylephrine and ephedrine groups. Consistent with our clinical observations. phenylephrine injection can lower the HR. Bradycardia in the elderly can be attributed to abnormal sinus node function, heart block, and myocardial ischemia. Therefore, elderly patients being administered phenylephrine should be carefully observed for adverse effects.

We observed sublingual microcirculation changes in elderly patients undergoing laparoscopic rectal cancer surgery. This observation may have resulted from (1) vasoplegia caused by anesthetic drugs, such as propofol and sufentanil, (2) the decreased vascular compliance of elderly patients, and (3) changes in arterial tone due to fasting and enema, which result in the loss of body fluids and electrolytes.

This observational study has several limitations. Although a blinded investigator conducted the retrospective microvascular analysis, a non-blinded investigator was responsible for video acquisition. Furthermore, the use of only ephedrine and phenylephrine in the study limits the generalizability of our findings as other vasopressors and fluid therapy may affect cerebral blood flow and sublingual microcirculation differently (31, 32).

## Conclusion

Elderly patients who underwent laparoscopic rectal cancer surgery and had MAP decline accompanied with sublingual microcirculation changes. Sublingual microcirculation was successfully restored by phenylephrine and ephedrine administration. Accordingly, based on our findings, it is recommended that ephedrine or phenylephrine should be immediately administered to improve MAP and avoid reduced microcirculation. In the future, microvascular sublingual parameters could be implemented as additional indicators of microcirculation.

## Data availability statement

The original contributions presented in this study are included in the article/supplementary material, further inquiries can be directed to the corresponding author.

## Ethics statement

The studies involving human participants were reviewed and approved by the Ethical Review Board of our hospital (The First Affiliated Hospital of Soochow University No. 2019130). The patients/participants provided their written informed consent to participate in this study.

## Author contributions

YZ and FJ designed and performed most of the investigation. LJ analyzed the data and wrote the manuscript. XM contributed to interpretation of the data and analyses. HL contributed to revising the article and modifying the figures. All authors have read and approved the manuscript.
